# Mortality and Life Expectancy in Homeless Men and Women in Rotterdam: 2001–2010

**DOI:** 10.1371/journal.pone.0073979

**Published:** 2013-10-02

**Authors:** Wilma J. Nusselder, Marcel T. Slockers, Luuk Krol, Colette T. Slockers, Caspar W. N. Looman, Ed F. van Beeck

**Affiliations:** 1 Department of Public Health, Erasmus MC, University Medical Center Rotterdam, Rotterdam, The Netherlands; 2 Municipal Public Health Service (GGD) Rotterdam-Rijnmond, GGD Rotterdam-Rijnmond, Rotterdam, The Netherlands; Universidade Federal do Acre (Federal University of Acre), Brazil

## Abstract

**Background:**

Data on mortality among homeless people are limited. Therefore, this study aimed to describe mortality patterns within a cohort of homeless adults in Rotterdam (the Netherlands) and to assess excess mortality as compared to the general population in that city.

**Methods:**

Based on 10-year follow-up of homeless adults aged ≥ 20 years who visited services for homeless people in Rotterdam in 2001, and on vital statistics, we assessed the association of mortality with age, sex and type of service used (e.g. only day care, convalescence care, other) within the homeless cohort, and also compared mortality between the homeless and general population using Poisson regression. Life tables and decomposition methods were used to examine differences in life expectancy.

**Results:**

During follow-up, of the 2096 adult homeless 265 died. Among the homeless, at age 30 years no significant sex differences were found in overall mortality rates and life expectancy. Compared with the general Rotterdam population, mortality rates were 3.5 times higher in the homeless cohort. Excess mortality was larger in women (rate ratio [RR] RR 5.56, 95% CI 3.95–7.82) as compared to men (RR 3.31, 95% CI 2.91–3.77), and decreased with age (RR 7.67, 95% CI 6.87–8.56 for the age group 20–44 and RR 1.63, 95% CI 1.41–1.88 for the age group 60+ years). Life expectancy at age 30 years was 11.0 (95% CI 9.1–12.9) and 15.9 (95% CI 10.3–21.5) years lower for homeless men and women compared to men and women in the general population respectively.

**Conclusion:**

Homeless adults face excessive losses in life expectancy, with greatest disadvantages among homeless women and the younger age groups.

## Introduction

Homeless people generally have substantial health disadvantages as compared to the general population [1]–[15]. Also, studies on the homeless have reported a high proportion of psychiatric disorders [16] and substance use [2], [10], as well as excess mortality related to different types of substance abuse [2], [10]. Moreover, homeless people have a higher prevalence of morbidity, and homelessness itself appeared to be an independent risk factor for mortality from specific causes, including drug-related conditions, circulatory diseases and respiratory diseases [9]. Studies have also documented variations in mortality within the homeless population, indicating higher mortality of whites as compared to other racial groups, which differs from that in the general population [15]. Also, among women, only older women retained the usual female survival advantage over their homeless male counterparts, whereas younger homeless women did not [3].

Only a few countries have investigated (excess) mortality of homeless people and most have been limited by small sample sizes, loss-to-follow-up and uncertain linkage of data [10]. Excluding studies which deal with a specific group of homeless people, e.g. persons in convalescence care [13], [17], persons with mental problems [18]–[20] or rough sleepers [12], mortality among the homeless has only been studied in the USA (Philadelphia [4], Boston [7], [21], [22], New York [1]), Canada (Toronto [3], [5], Montreal), Denmark (nationwide [10], Copenhagen [11]), Sweden (Stockholm [2], [18]), and the United Kingdom (Glasgow [9]). Of these, only 4 described mortality of homeless cohorts in the 21^st^ century [2], [9], [10], [15]. Another limitation of earlier studies is that they often exclude the most vulnerable groups, such as those sleeping rough.

This study aims to describe mortality patterns by age, sex, and type of service use within a cohort of homeless people and to assess excess mortality as compared to the general population of Rotterdam between 2001 and 2010. The goal was to investigate a comprehensive selection of the homeless by including homeless people in contact with very diverse types of services, ranging from only meal services to night-care facilities and convalescence care, and by including services provided by local authorities and by charitable organizations. Based on almost 10 years follow-up, we describe mortality differentials within the homeless cohort, and between the homeless and the general population. Life table decomposition analysis is used to assess to what extent different age groups contributed to the disparity in life expectancy between the homeless and the general population.

## Methods

### Ethics statement

The Medical Ethical Review Committee of the Erasmus MC declared that this study was not subject to the Law on Medical Research with human beings and that it had no objections to the performance of this study.

### Study population

#### Cohort of homeless people

Institutions providing care to homeless people in Rotterdam were approached to provide full name, date of birth and sex of persons who visited their facility in 2001. This information was largely available because in 2001 homeless people were counted during research on the homeless in Rotterdam [23]. Institutions subsidized by the local government and services provided by the church were included, covering services at different locations in Rotterdam. These care facilities provide many services to homeless people (including those sleeping rough), ranging from the provision of meals, to night-care facilities and convalescence care for ill homeless. Pooling the lists from the different facilities yielded 5810 records: removal of duplicates with same name, initials/surname, date of birth and sex yielded 3398 persons.

The study on Rotterdam homeless conducted in 2001 counted 4607 homeless people [23].

The difference between this higher number compared with our cohort of 3398 persons may be because non-Dutch EU citizens and illegals not in the Dutch municipal population registers may have used care services in 2001, and the 2001 total of 4607 persons may have included duplicates. In the present study, the deletion of duplicates may have been more efficient due to the availability of several computerized registries.

We used a restrictive matching procedure to ensure that for all matched persons we could determine in the municipal population registers whether (and if so when) that person had died. Of the 3398 persons in our cohort, 2159 provided a match with the municipal population registers (which include information on vital status and date of death). A total of 7 persons had died before the start of the follow-up. In addition, we excluded persons aged under 20 years to allow comparison with administrative data in 5-year age groups (e.g. 20–24 years) and to avoid the small numbers of death below age 20 years. Finally, our study population consisted of 2096 persons.

Based on the lists provided by the care facilities in 2001, homeless persons were classified as “only day care”, “convalescence care” and “other” (i.e. sleeping rough, only night care, day and night care). Day care is a walk-in facility for social support, medical care, activities, etc. Convalescence care is a medical care unit providing 24-h care where the homeless stay for a shorter or longer period with an indication for somatic care; however, homeless persons with psychiatric and/or addiction problems are also admitted. Night care is a service that provides a ‘bed and bread’ for one night for persons with no place to stay; each day, the homeless have to register for the following night. ‘Sleeping rough’ indicates persons who, during the whole year, sleep several times per month outside. This includes people sleeping in squats, or shacks/garden houses/sheds, etc. without gas, electricity and water.

Risk time was calculated from the number of days between study entry in 2001 and date of death, or 1 December 2010, or date of loss-to-follow up, whichever came first. Study entry was assumed to be on July 1 2001 (i.e. halfway 2001). Only for people who died in the first six months of 2001 was follow-up time assumed to have started halfway between January 1 2001 and the date of death. In the municipal population registries, 242 persons were classified as ‘emigrated’, which is an administrative term for ‘loss to follow-up in the administration’. Of these, 90 could be traced in other registers (i.e. the GP register “microHIS”, Health insurance system, or Municipal OGGZ system) and were included in the follow-up time until the last date they were present in at least one register. The remaining persons were assumed to have been at risk until halfway the follow-up period.

#### General population of Rotterdam

Data for our homeless cohort were compared with the general population of Rotterdam region, aged 20 years and over. Data on the mid-year population and number of deaths by sex, and 5-year age groups were derived from Statline (http://statline.cbs.nl/statweb/, accessed March 29, 2011) for the period 2001–2009.

### Statistical methods

Poisson regression was used to assess the association of mortality among homeless with age and sex and their interaction, and with the type of service used corrected for age and sex. For this purpose, follow-up time of the homeless people was split into one-year periods of follow-up time by age (in complete single-years), and individual characteristics (sex, type of service use) were added. Poisson regression estimates the observed numbers of death by a multiplication of person years and a mortality rate that is the exponent of a linear predictor (alpha + beta_1×_1+ beta_2×_2+ beta_3×_3…). For a model with age and gender the linear predictor consists of two categorical variables operationalized by dummy x1 for being female (male is the baseline) and several dummies for age group: x2 being in the second age group (youngest age group is baseline), x3 etc. Exp(alpha) is the rate for males in the youngest (baseline) age group. The effect of each variable is corrected for the other variables. We did not correct for period, as mortality did not differ significantly within the 10-year of follow up. Poisson regression yields rate ratios (RRs) which in our model indicate the increase/decrease in the mortality rate relative to the reference category (e.g. homeless women as compared to homeless men). Age was grouped in 10-year age groups. Poisson regression with follow-up time by calendar year and age yields the same results as Cox regression with age as baseline hazard if the variable age is truncated to 10-year age groups [24]–[26]. Poisson regression including age*gender interactions yields the same results as an age-sex stratified analyses, but also provides overall p-values for the significance of the interaction.

Next, we compared mortality between the homeless and the general Rotterdam population. We first constructed a similar dataset for the homeless and general Rotterdam population with person-years and deaths by 5-year age group and sex for the period 2001–2010. Therefore, we aggregated person-years at risk and deaths among the homeless population across ages into 5-year age groups. Data on the general Rotterdam population were obtained from Statline and had the same format.

Poisson regression with person-years as rate multiplier was used to assess the association between homelessness and mortality, and to assess whether this association differed by age and sex. This yields RRs comparing mortality rates among homeless men with those among men in the general population, corrected for age. The same model yields age-specific RRs, corrected for gender. These RRs can be interpreted as standardized mortality ratios [27], and indicate the amount of excess mortality in the homeless population relative to the general population. We calculated overall RRs corrected for age (5-year age groups) and sex, gender-specific RRs corrected for age, and age-specific RRs corrected for gender. To increase the power we defined these interactions between homelessness and age using broader age groups (<45, 45–59, 60+ years).

Finally, we used the actuarial method to calculate remaining life expectancy by sex for homeless men and women, and for men and women in the general population for different starting ages. Life expectancies are not affected by the population structure and therefore can be directly compared between populations or population groups. Confidence estimates were obtained using the method proposed by Chiang [28]. To further examine the difference in life expectancy between the homeless and the general population, we assessed the contribution of different age groups to the disparity in remaining life expectancy using the life table decomposition method developed by Arriaga [29]. This method partitions the total difference in life expectancy between two populations or time periods into age-specific contributions. The method takes into account the effect of a difference in mortality in a specific age group on the number of person-years in that age group and in all older age groups. The Arriaga method is frequently used to decompose differences in life expectancy. Confidence intervals (CIs) for the contributions by age were calculated using parametric bootstrap, assuming a Poisson distribution around the observed numbers of deaths. This method gives problems if the observed number is zero (the variance is then also zero) as was the case for homeless women aged 20–30 years. Therefore, we added results for remaining life expectancy at age 30 years. The program for decomposition of life and health expectancy [30] is available from the authors on request.

## Results

### Mortality in the homeless cohort

The present cohort includes 2096 homeless adults aged ≥ 20 years in 2001. Table 1 presents their characteristics: there were 1846 men and 250 women with a mean age of 40.6 years. A total of 17,619 person-years were observed, with a mean follow-up of 8.4 years. In total, 265 homeless persons (232 men and 33 women) died during follow-up. Table 2 presents the number of person-years, deaths and mortality rates of the homeless men and women by age.

**Table 1:** Characteristics of the cohort of homeless men and women in Rotterdam, 2001.

Characteristic	No. (%)
**Age in years**	
Mean	40.6
20–29	345 (16)
30–39	711 (34)
40–49	665 (32)
50–59	260 (12)
60–69	89 (4)
70+	26 (1)
**Sex**	
Men	1846 (88)
Women	250 (12)
**Mean duration of follow-up (years)**	8.4
**Type of service**	
Only day care	635 (30)
Convalescence care	80 (4)
Other (night care, day care + night care, sleeping rough)	1381 (66)
Deaths during follow-up	266 (13)
Emigrated (i.e. lost to follow-up)	248 (12)
Alive at 1 November 2011	1588 (76)

**Table 2:** Age-specific person-years, deaths, and mortality rates of homeless men and women, Rotterdam, 2001–2010.

	Men			Women		
Age group (years)	Person-Years	Deaths	Mortality rate per 100 000 (95% CI)	Person-Years	Deaths	Mortality rate per100 000 (95% CI)
20–29	1427	13	911 (529–1569)	235	0	0
30–34	4372	34	778 (556–1088)	773	8	1035 (518–2069)
40–49	5539	65	1173 (920–1496)	737	15	2037 (1227–3276)
50–59	2977	62	2083 (1624–2671)	220	5	2274 (946–5460)
60–69	939	32	3408 (2410–4819)	83	2	2396 (603–9635)
70+	262	26	9935 (6757–14575)	55	3	5476 (1759–16913)

Within the homeless population, increasing age was associated with an increase in mortality risk (p<0.001). After correction for age, being female was not associated with mortality within this cohort (p = 0.55); the RR for males vs. females was 1.12 (95% CI 0.77–1.61). The interaction between age and sex was not significant (p = 0.12). The effect of type of service used (only day care services, convalescence care, other), corrected for age and sex was borderline significant (p = 0.074). Relative to persons using day care only in 2001, persons using convalescence care (and possibly other services) had a 70% higher mortality rate (RR 1.73, 95% CI 1.04–2.88).

### Mortality in the homeless population compared with the general Rotterdam population

In the general Rotterdam population the RR for females vs. males, corrected for age, was 0.67 (95% CI 0.66–0.69), which is markedly different from the RR in the homeless population (1.12, 95% CI 0.77–1.61).

Homeless men and women in Rotterdam had a 3.5 times higher mortality rate as compared to their peers in the city's general population (Table 3). The disparity in mortality between homeless and non-homeless people was greater for women (RR 5.56) than for men (RR 3.31) (p = 0.002 for the interaction between gender and homelessness). The excess mortality varies by age (p<0.001 for the interaction between age and homelessness). The RR decreased from 7.67 for the age group 20–44 years to 1.63 for the age group 60+ years.

**Table 3:** Rate ratios when comparing mortality rates in the homeless population with the general Rotterdam population.

	Rate ratios (95% CI)	p-value
**Within homeless population**		
Age (corrected for sex)		<0.001
<30	1	
30–39	1.04 (0.56–1.94)	
40–49	1.63 (0.91–2.94)	
50–59	2.70 (1.49–4.90)	
60–69	4.28 (2.26–8.12)	
70+	11.67 (6.07–24.45)	
Female sex (corrected for age)	1.12 (0.77–1.61)	0.550
Service use		0.074
Only day	1	
Convalescence care	1.73 (1.04–2.88)	
Other (day and night, only night,sleeping rough)	0.93 (0.72–1.22)	
**Homeless vs. general population**		
Total (corrected for age and sex)	3.49 (3.09–3.94)	<0.001
Gender corrected for age		0.006
Men (corrected for age)	3.31 (2.91–3.77)	<0.001
Women (corrected for age)	5.56 (3.95–7.82)	<0.001
Age in years (corrected for sex)		<0.001
20–44	7.67 (6.87–8.56)	<0.001
45–59	3.70 (3.41–4.01)	<0.001
60+	1.63 (1.41–1.88)	0.001

### Life expectancy of homeless men/women and of men/women in the general Rotterdam population

Remaining life expectancy at age 20 years was 43.1 (95% CI 39.8–46.4) years for homeless men and 46.2 (95% CI 40.6–51.8) years for homeless women (Table 4). At age 30 years, the remaining life expectancies of homeless men and women were 36.7 (95% CI 34.8–38.6) and 36.2 (95% CI 30.6–41.8) years, respectively. At both 20 and 30 years, the difference in life expectancy between homeless men and women was not significant.

**Table 4:** Remaining life expectancy and differences in remaining life expectancy at age 20 and 30.

	At age 20,in years (95% CI)	At age 30,in years (95% CI)
**Remaining Life expectancy**		
Homeless men	43.1 (39.8–46.4)	36.7 (34.8–38.6)
Homeless women	46.2 (40.6–51.8)	36.2 (30.6–41.8)
		
Rotterdam men	57.4 (57.3–57.5)	47.7 (47.6–47.8)
Rotterdam women	61.9 (61.8–62.0)	52.1 (52.0–52.2)
**Difference in remaining life expectancy**		
Homeless men – Homeless women	−3.1 (−9.6–3.4)	0.5 (−5.4–6.4)
Homeless men – Rotterdam men	−14.3 (−17.6–11.0)	−11.0 (−12.9–−9.1)
Homeless women – Rotterdam women	−15.8 (−21.4–−10.2)	−15.9 (−21.5–−10.3)

Life expectancy of homeless men and women was significantly lower than among men and women in the general Rotterdam population. At age 20 years, life expectancy of men in the general Rotterdam population was 57.4 (95% CI 57.3–57.5) years and of women 61.9 (95% CI 61.8–62.0) years, i.e. 14.3 (95% CI 11.0–17.6) and 15.8 (95% CI 10.2–21.4) years higher than that of homeless men and women, respectively. At age 30 years this difference was 11.0 (95% CI 9.1–12.9) and 15.9 (95% CI 10.3–21.5) years, respectively.

**Figure 1:**
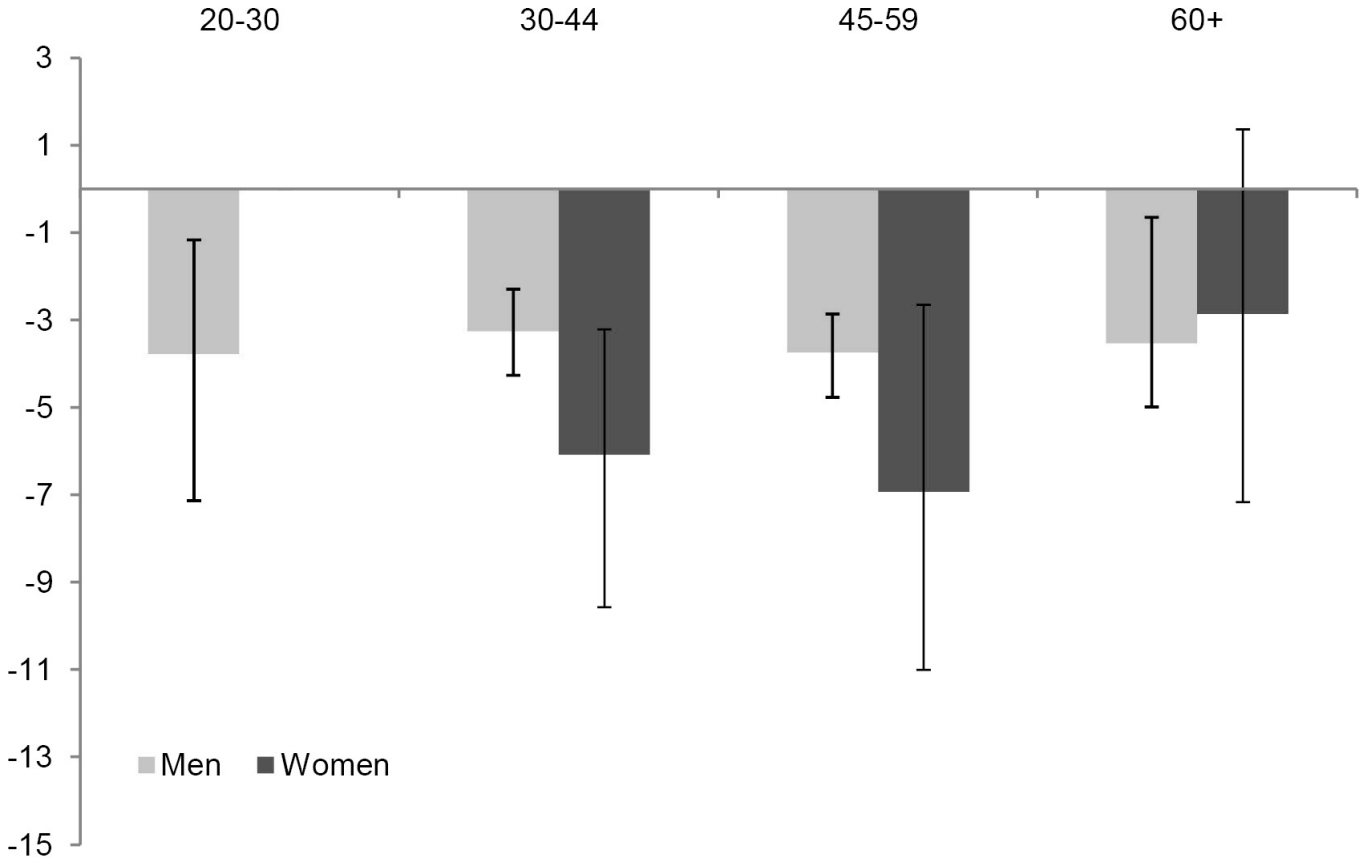
Contributions of age groups to differences in remaining life expectancies of homeless men and women as compared to the general Rotterdam population of men and women. Difference in remaining life expectancy (homeless population – Rotterdam population) of men and women, at age 20 years^§^ decomposed by age group (in years). ^§^ For women the contribution below age 30 years is not presented because the number of deaths in homeless women was zero and the confidence intervals could not be obtained in the situation of zero deaths.

Figure 1 shows the contribution of the different age groups to the disparity in remaining life expectancy between the homeless and the general population.

Compared with men in the general Rotterdam population, the 14-year lower life expectancy of homeless men aged 20 years was due to higher mortality among homeless men in all four age groups. Each age group contributed about 3.5 years to the total gap of 14 years in remaining life expectancy at age 20 years. All age groups (except for the youngest age group) contributed almost equally to the total gap of 11 years in remaining life expectancy at age 30 years.

Compared with the general population, the 16-year lower life expectancy of homeless women aged 30 years was due to large losses between age 30–44 and 45–59 years (each explaining about 6 years of the gap) and smaller losses at age 60+ years (explaining about 3 years of the gap).

## Discussion

Mortality rates among the homeless increase with age, but are not consistently lower in homeless females compared with homeless males. Mortality among the homeless is 3.5 times higher than among the general population of Rotterdam. The greatest disadvantages, as compared to the general population, were found for homeless women and the younger age groups. In homeless women mortality was more than 5 times higher and in homeless men 3 times higher. For the age groups 20–44 years, mortality was almost 8 times higher and about 1.5 times for the age group 60+ years. Remaining life expectancy of homeless people at age 30 years was substantially lower compared with Rotterdam's general population: a disparity of 11 years for men and 16 years for women.

Our study confirms large excess mortality among homeless people as compared to the general population, and variations in the excess mortality with age, generally showing higher excess risks at younger ages [1], [4], [31]. This study extends the available evidence from a limited number of countries by including data from the Netherlands, with results on mortality and life expectancy of a comprehensive homeless cohort from Rotterdam. Another study performed in the Netherlands also investigated mortality of homeless people, but focused on homeless in convalescence care only [13]. Moreover, our study examined mortality in the period 2001–2010, thereby extending and updating the evidence base.

Our RRs of 3.3 (men), 5.6 (women), 3.5 (both sexes combined) are in line with other studies reporting RRs ranging from 3.1 [2] to 5.6 [10] in men, from 2.5 [2] to 6.7 [10] in women, and 4.4 [9] for both sexes combined. Thus, the present study provides the evidence that in the 21^st^ century homelessness is still associated with a large excess mortality, even in a country like the Netherlands with an extensive social security system. The high excess mortality in the current century is in line with a recent study reporting that in 2003–2008 the mortality rate of 18–64 year-old homeless in Boston has not changed as compared to 1988–1993 [15].

Previous studies comparing mortality among homeless men and women report inconsistent results, which may (in part) reflect the use of different outcome measures and/or statistical analyses and/or study populations, and perhaps variations by age. However, similar to our study, some studies found no significant overall difference in mortality between homeless men and women [2], [11], whereas others found lower mortality and higher life expectancies in homeless women [10], [32]. A study based on 7 cities found that mortality rates among younger homeless women often approach or equal those of homeless men, but were lower in females at older ages [3]. Similar to our study, most other comparisons of mortality rates between homeless men/women and men/women in the general population, showed higher excess mortality in women than in men [1], [5], [7], [10], [11]. An exception was a study among Canadian homeless and marginally housed people which reported smaller standardized mortality ratios and a smaller gap in life expectancy when comparing women living in shelters, rooming houses and hotels with the entire cohort [32]. Strikingly, a large register-based study in Denmark also found a smaller gap in life expectancy of homeless as compared to the general population of women [10], which is in contrast to the higher overall standardized mortality rate for females reported in the same study. Similar to most other studies, including the Danish study [10], we found higher excess mortality in women in terms of mortality RRs; however, in our study this was confirmed by a larger disparity in life expectancy as compared to the general population in women.

Mortality RRs and life expectancy measures summarize mortality across age. Differences between these aggregated measures may yield less interpretable outcomes if mortality variations differ by age. For life expectancy this is widely recognized and life table decomposition analysis is often used to provide insight into the contributions of different age groups [29]. In the present study, the decomposition of the disparity in life expectancy at age 30 years between the homeless and the general Rotterdam population by age showed particularly for women large contributions of the age groups 30–44 and 45–59 years to the gap in life expectancy. Decomposition analyses may provide a better understanding of the inconsistent gender differences in excess mortality in the Danish study [10].

Our study includes homeless people in contact with a wide range of services (i.e. persons who use only day care, only night care, both night and day care, convalescence care, and sleeping rough). Homeless in convalescence care had about 70% higher mortality compared with homeless persons using only day care (Table 3) and 80% higher compared with all other services combined (data not shown). The higher mortality of homeless in convalescence care may well be a selection effect. Therefore, to avoid underestimation of mortality among the homeless, it is important to include this vulnerable latter group in the study population.

Our study has some limitations. First, homeless people who did not use any local service in 2001 were not included, and not all institutes could provide data for 2001. In addition, the present study lacks data on 130 addicted female sex workers and about 225 persons living in social pensions. Female sex workers may have a higher mortality rate than the women included in our study. Also, one institution provided a list that was based on their 2001 administration but was actually compiled at a later date.

Another limitation is that we were unable to link (a maximum of) 1238 homeless who were on the lists in 2001. In the Netherlands, in 2001 the ‘citizen service number’ (BSN; a unique identification number) was not yet legally required and was not used in the lists. We cannot rule out that specific groups with a higher mortality rate were more likely to be missed. Also, differences and/or errors in spelling are more likely to occur with non-Dutch names. Homeless immigrants without a legal residence status who were not later legalized could by definition not be matched and may have faced worse living circumstances than their legal homeless peers. On the other hand, the 1238 records that could not be matched is probably an overestimation of the number of unmatched persons as it may include duplicates and persons already included in the study cohort. The strict matching approach guaranteed that we had almost complete information on the vital status throughout the study follow-up. Only for 242 persons registered as ‘emigrated’ was the follow-up not complete; however, excluding these latter persons did not change the study conclusions.

We have no data on whether those homeless in 2001 were still homeless during follow-up. Some probably succeeded in acquiring housing, which may have led an underestimation of mortality related to homelessness. However, this limitation does not hamper our comparison with other studies, as almost all studies assessed the effect of homelessness at the start of follow-up.

Our study cohort was relatively small, with comparatively few women (n = 250). This is partly compensated by the relatively long follow-up period; nevertheless, our results based on data for women should be interpreted with caution (especially since data on 130 addicted female sex workers were not included). Another limitation is that our study does not provide information on cause of death or on any other characteristic (apart from age, sex and type of service use).

Based on almost 10 years of follow-up, for a representative cohort of homeless people this study presents: 1) mortality RRs comparing mortality among demographic subgroups of the homeless, 2) mortality RRs comparing mortality between homeless men and women and men and women in the general Rotterdam population, and 3) life expectancy differences for men and women between the homeless and the general Rotterdam population. The comparisons among the homeless population, and between the homeless and the general population, provide some useful insights which complement each other. Large excess mortality indicates high losses associated with homelessness and shows that large potential gains might be achieved by preventing persons from becoming homeless, or by reducing the adverse health effects associated with being homeless. The adverse health effects appear to be greater for women. This higher excess mortality may reflect that younger homeless women, compared with homeless men, practice worse health behaviors [17], [23], have a higher risk of alcohol/drug use disorders [17] and have a higher risk for psychiatric disorders [17]. Younger homeless women have been found to have a significant higher risk for hospital admission; risks were considerably elevated for diseases of the genital organs, viral hepatitis, and poisoning [33]. A recent meta-synthesis study of Finfgeld-Connett [34] describes the downward spiral of women becoming homeless and the role of complex interconnected stressors, the non-adaptive attachments with men, and flawed problem-solving and decision-making skills of homeless women in their life as homeless. Homelessness is much less frequent among women [33], [35] and hence homeless women may be more negatively selected than their male peers. But apart from selection processes also causation processes may play explain the even worse health situation of homeless women. Homeless, women may be more vulnerable to the adverse health effects of the social environment [33] and may experience more barriers to care [35]. The most vulnerable groups need to be reached, including women with mental health problems and excessive use of alcohol/drugs, for instance by better aftercare following detention or admission to psychiatric care. However, in general the overall excess mortality among the homeless is high. This also suggests the need for a broad approach. For example, in 2006 the Dutch government, together with the four major Dutch cities (Amsterdam, Rotterdam, The Hague and Utrecht) started a program to guide all homeless persons into temporary care and social rehabilitation and, subsequently, into clustered or individually supported housing projects, with social education training and guidance into jobs.

## Conclusion

In this century, and even in the Netherlands with its relatively extensive social safety nets, homelessness is associated with substantially higher mortality and a dramatic loss in life expectancy. The disparity in mortality between homeless people and the general Rotterdam population is greatest for women and younger persons. Subsets of the general population who tend to have a survival advantage (i.e. females and younger ages groups) are those who are most adversely affected by homelessness.
